# Supplementary material for: The Tudor protein Veneno assembles the ping-pong amplification complex that produces viral piRNAs in Aedes mosquitoes
Source: Nucleic Acids Res. 2018 Dec 19;47(5):2546–59. doi: 10.1093/nar/gky1266 (PMC6411831; doi:10.1093/nar/gky1266)
Supplement: Supplementary Data [file gky1266_supplemental_files.pdf]

**SUPPLEMENTAL INFORMATION**

for

# The Tudor protein Veneno assembles the ping-pong amplification complex that produces viral piRNAs in *Aedes* mosquitoes

**Joep Joosten[1], Pascal Miesen[1], Ezgi Taşköprü[1], Bas Pennings[1], Pascal W.T.C. Jansen[2], Martijn A. Huynen[3], Michiel Vermeulen[2], and Ronald P. Van Rij[1+]**

[1]Department of Medical Microbiology, Radboud Institute for Molecular Life Sciences, Radboud University Medical Center, P.O. Box 9101, 6500 HB Nijmegen, The Netherlands
[2]Department of Molecular Biology, Faculty of Science, Radboud Institute for Molecular Life Sciences, Oncode Institute, Radboud University Nijmegen, P.O. Box 9101, 6500 HB Nijmegen, The Netherlands
[3]Centre for Molecular and Bioinformatics, Radboud Institute for Molecular Life Sciences, Radboud University Medical Center, P.O. Box 9101, 6500 HB Nijmegen, The Netherlands

[+] corresponding author: ronald.vanrij@radboudumc.nl

# Supplemental Figure 1.

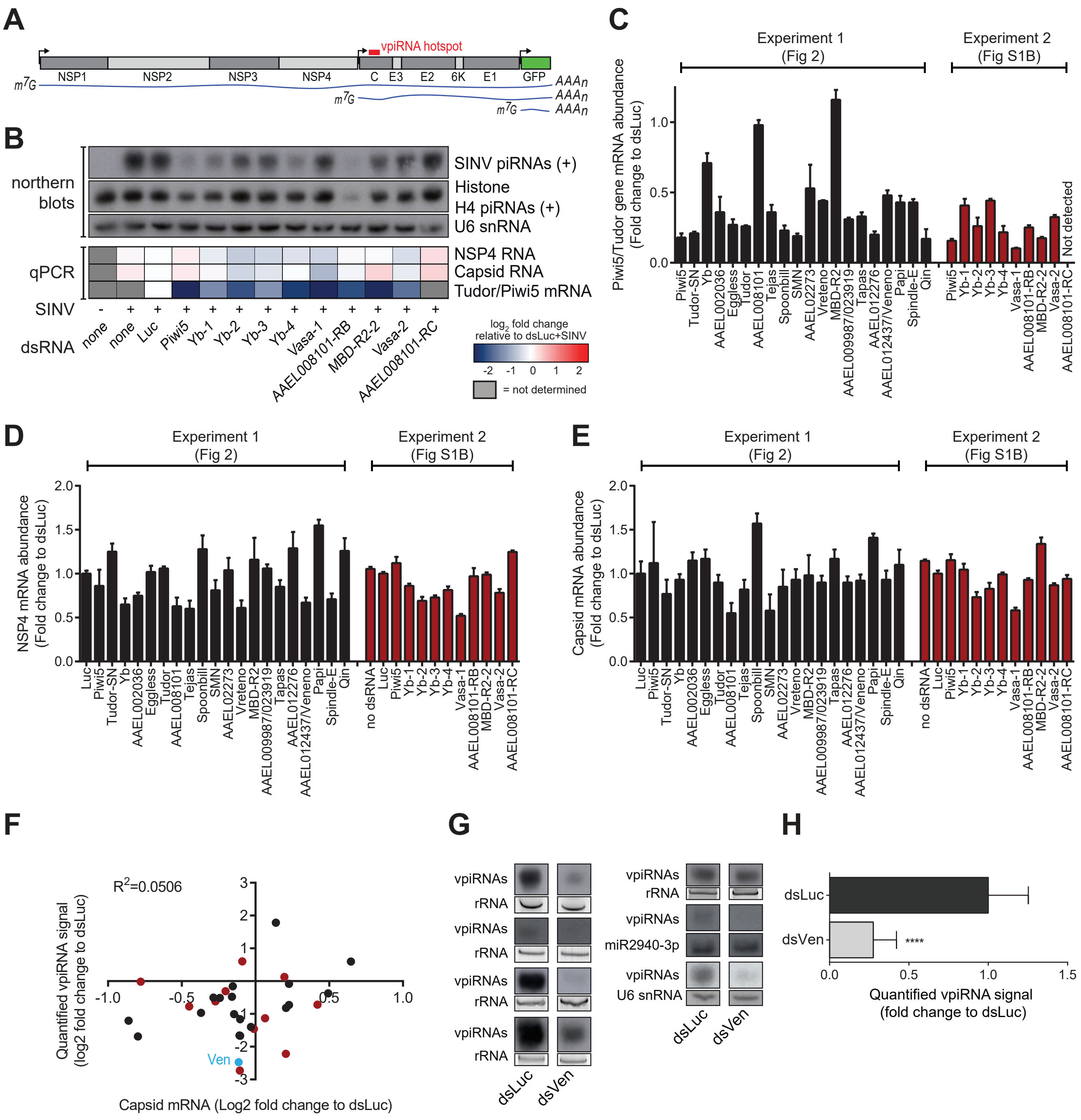


**Figure S1. Viral RNA levels are unaltered upon knockdown of Tudor genes.**
**(A)** Schematic representation of the SINV-GFP genome. Individual viral proteins are depicted in dark and light grey and the GFP expressed under a duplicated sub-genomic promoter is shown in green. The blue lines represent the (+) strand RNA species that are produced during the course of an infection. The red bar indicates the hotspot region in the Capsid gene that gives rise to the abundant vpiRNAs probed for in Figure 2 and S1B. **(B)** For genes with insufficient knockdown in the screen depicted in Figure 2, a second knockdown with different batches of dsRNA was performed. Numerical suffixes refer to dsRNA of different sequence (Yb 1-4 and Vasa 1-2), whereas AAEL008101-RB and -RC refer to the two splice variants of this gene. After knockdown, small RNA production of (+) strand Sindbis virus (SINV) and Histone H4 mRNA (H4)-derived piRNAs was assessed by northern blot. The heat map depicts relative changes in NSP4 and Capsid viral RNA levels and Tudor/Vasa/Piwi5 mRNA abundance as measured by RT-qPCR. **(C)** Relative abundance of targeted transcripts in the knockdown experiments shown in Figure 2 (experiment 1) and S1B (experiment 2). Expression levels were normalized to control samples treated with dsRNA targeting Firefly Luciferase (dsLuc). **(D-E)** Relative quantification of viral RNAs by RT-qPCR with primers located in the NSP4 (D) and Capsid (E) genes. Note that the Capsid sequence is present in both genomic and subgenomic RNA, whereas NSP4 is only present on genomic RNA. Bars in (C-E) are the mean +/- SD of three biological replicates. **(F)** No correlation was observed between the level of Capsid RNA expression as determined by RT-qPCR and the quantified vpiRNA signal on northern blots in Tudor knockdown experiments. Quantified vpiRNA signal was normalized to U6 snRNA signal, and expressed relative to dsLuc. Black and red circles correspond to the experiments shown in Figure 2 and S1B, respectively. The light blue circle indicates Veneno (Ven). The Pearson correlation coefficient (R2) was determined using GraphPad Prism 6. **(G)** Images of independent Ven-knockdown experiments used for signal quantification shown in (H); the vpiRNA signal is shown for each blot together with the signal used for normalization. Probing for miR2940-3p or U6 was used for normalization of two blots, whereas EtBr-stained rRNA served as loading control for the remainder. **(H)** Quantification of vpiRNAs produced upon Veneno knockdown (dsVen) and control knockdown (dsLuc). Bars are the mean +/- standard deviation of the quantified signals from the northern blots shown in (G), normalized to the dsLuc sample. Two-tailed student's T-test was used to determine statistical significance (****, $P = 8.82 \times 10^{-6}$).

## Supplemental Figure 2.

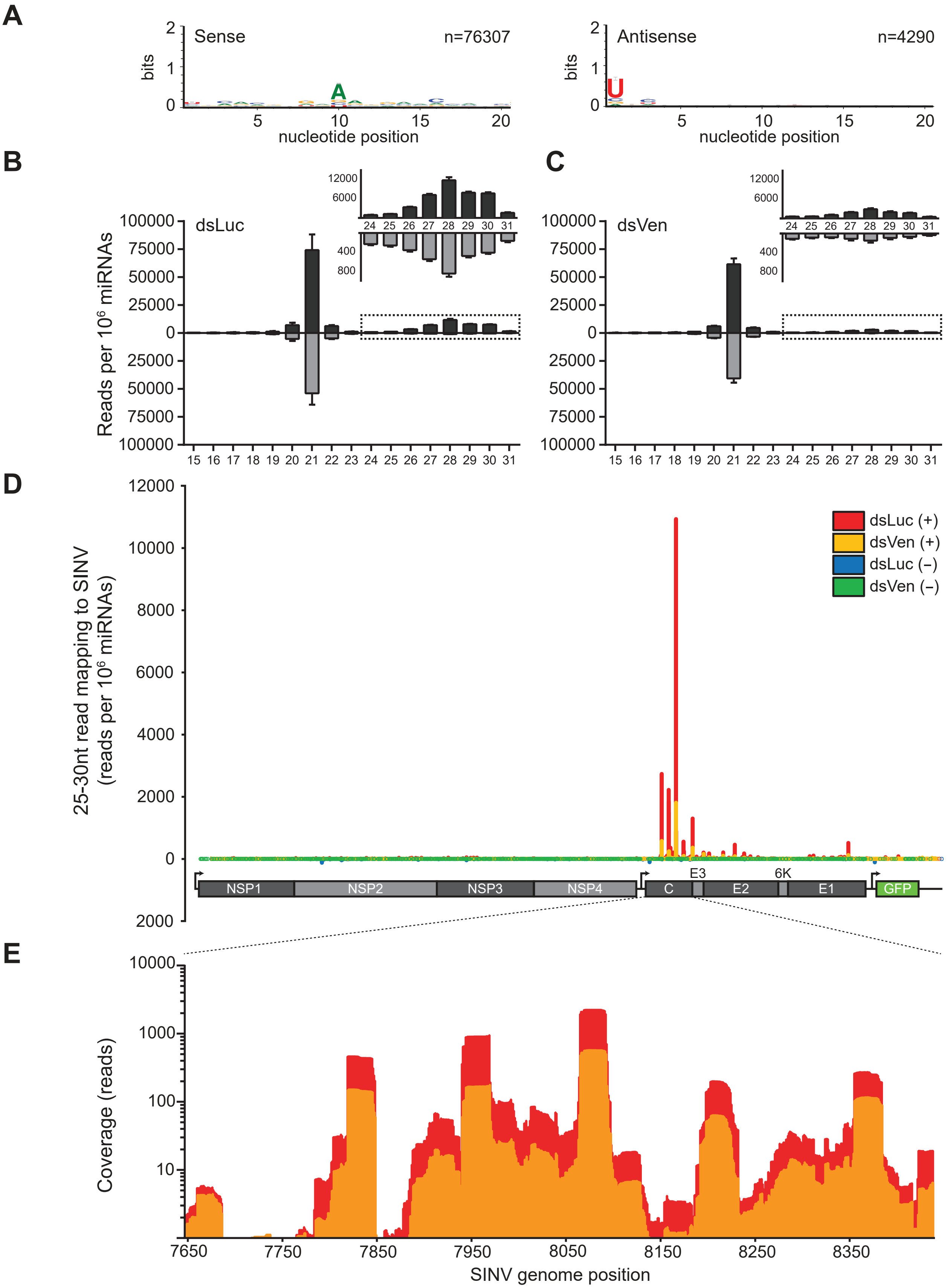


**Figure S2. Veneno depletion affects vpiRNA abundance, but not the vpiRNA profile.**
**(A)** Nucleotide bias at the first 20 positions in the 25-30 nt small RNA reads mapping to sense strand (left) and antisense strand (right) of the SINV genome in dsVen libraries (n = number of reads from three independent libraries combined). **(B-C)** Size profile of 15-31nt reads mapping to the sense (black) and antisense (grey) strands of Sindbis virus (SINV) in control (dsLuc, A) and Veneno knockdown (dsVen, B) libraries. The inset (traced by dashed line) shows piRNA sized (24-31 nt) reads in both libraries. Bars indicate the mean +/- SD of three independent libraries. **(D)** Distribution of 25-30 nt sense (dsLuc: red; dsVen: yellow) and antisense (dsLuc: blue; dsVen: green) reads across the SINV-GFP genome. The viral genome organization is depicted schematically below the graph. **(E)** Coverage of 25-30 sense reads across the vpiRNA hotspot region in the subgenomic region (nt 7647-8438) for both dsLuc (red) and dsVen (yellow).

# Supplemental Figure 3.

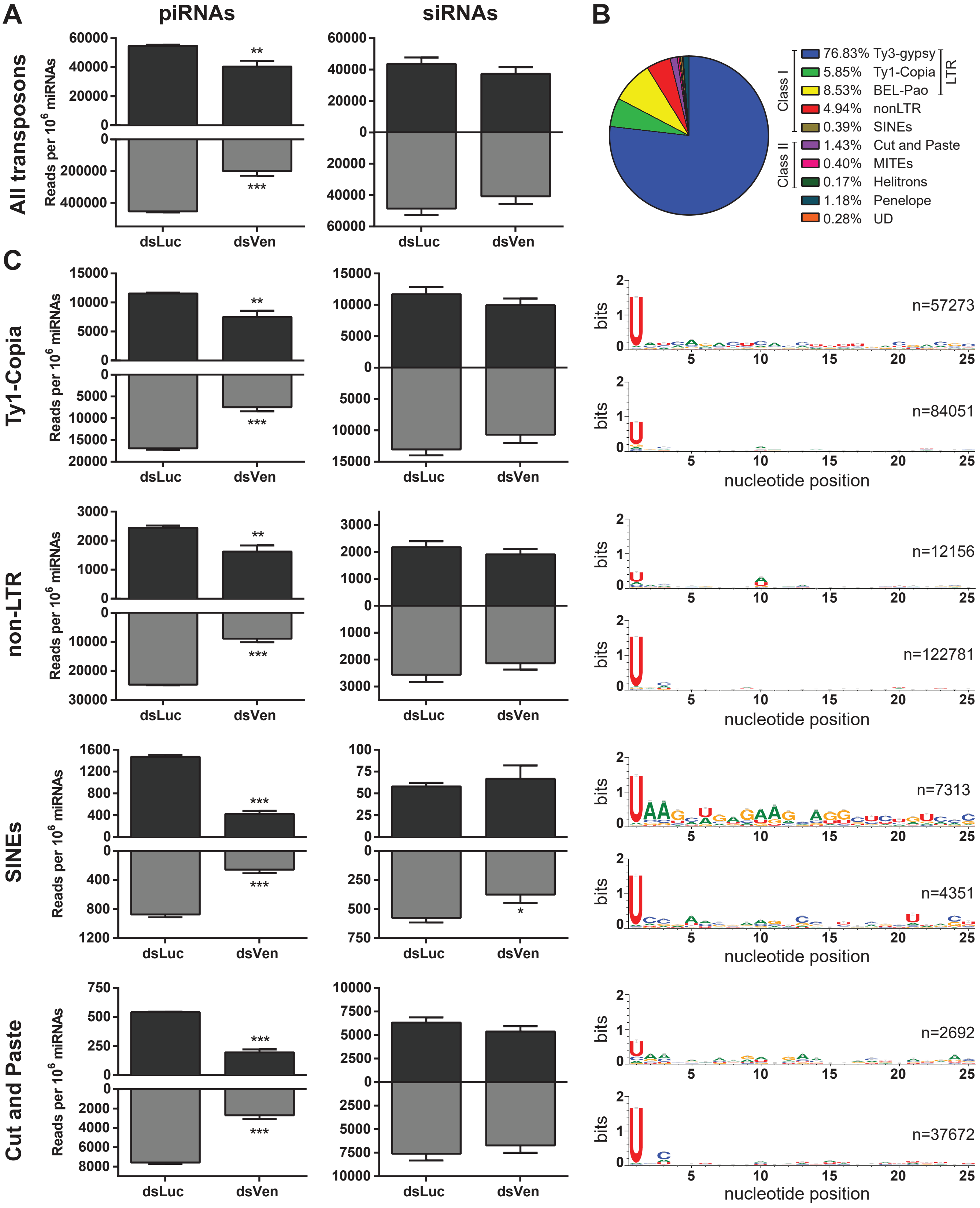

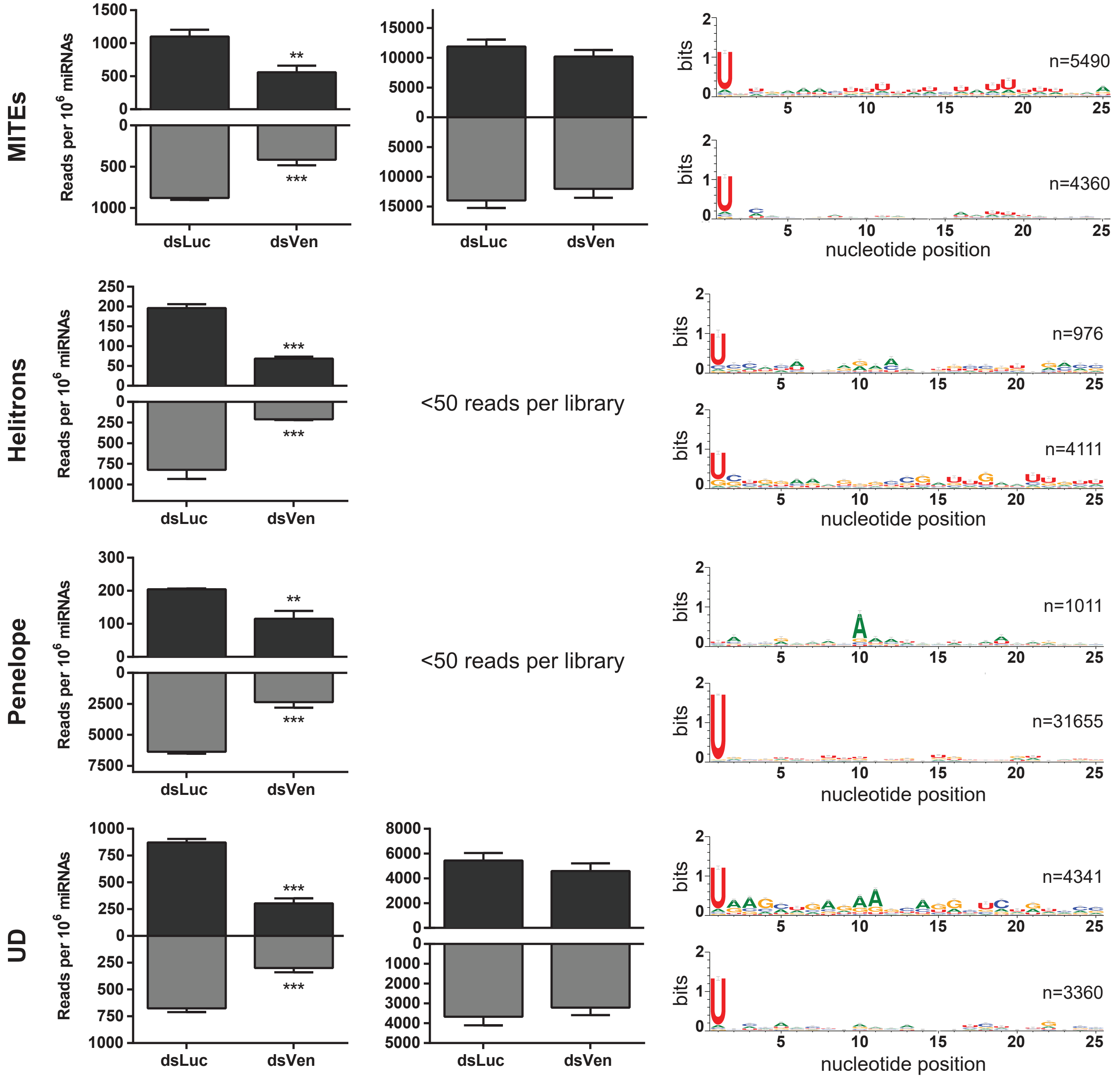


**Figure S3. Knockdown of Veneno differentially affects piRNA biogenesis from transposon subclasses.**
**(A)** The sum of normalized read counts of 25-30 nt piRNAs (left) and 21 nt siRNAs (right) mapping to all transposon families combined. **(B)** Pie-chart indicating the percentage of transposon piRNAs that map to different subclasses of transposable elements for all deep-sequencing libraries combined. The contribution of transposon subclasses to the piRNA population was indistinguishable between dsLuc and dsVen libraries. **(C)** The sum of normalized read counts of 25-30 nt piRNAs (left) and 21 nt siRNAs (middle) mapping to the transposon subclasses indicated on the left. Less than 50 siRNA-sized reads per library mapped to Helitron and Penelope elements and were therefore not plotted. Panels on the right show the nucleotide bias at the first 20 positions in the 25-30 nt small RNA reads mapping to sense strand (upper panel) and antisense strand (lower panel) in dsLuc control libraries for each transposon subclass. The bars are mean +/- standard deviation. Two-tailed student's T-test was used to determine statistical significance (* $P < 0.05$; ** $P < 0.01$, *** $P < 0.001$). To generate sequence logos, all reads from three independent dsLuc control libraries were combined (n=number of reads).

# Supplemental Figure 4.

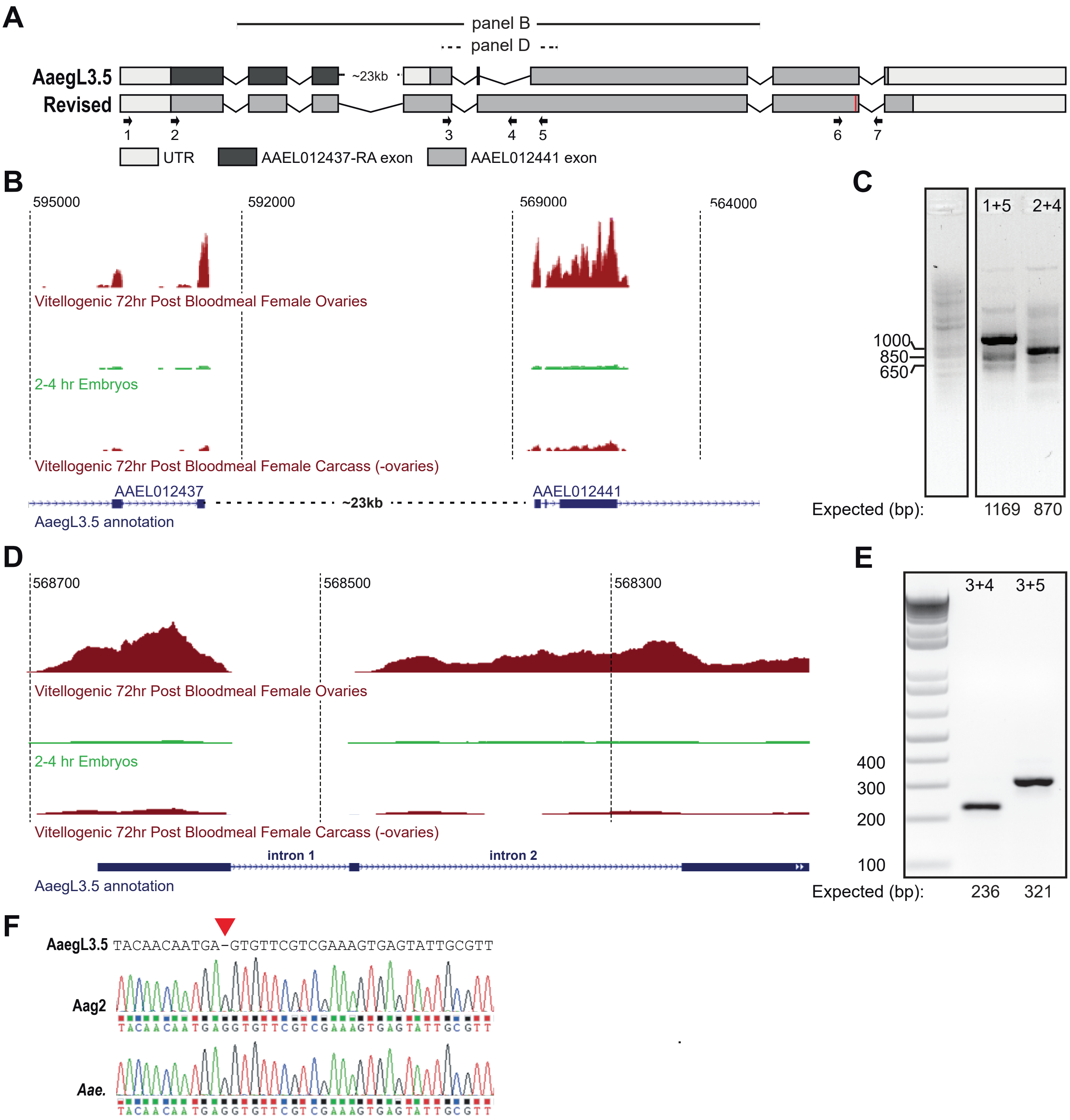


**Figure S4. Revised gene annotation of AAEL012441/Veneno (Changed to AAEL012437 in the current genome annotation - AaegL5.1)** **(A)** Schematic representation of the gene annotation for AAEL012441 as published by VectorBase (AaegL3.5, December 2017) and our revision based on sequencing of PCR fragments. In our revision, the sequences annotated as AAEL012437 and AAEL012441 in VectorBase are both part of the Veneno (Ven) transcript. Moreover, the sequence annotated in AaegL3.5 as the second intron of AAEL012441 is part of the coding sequence in our revised annotation. Finally, the carboxyl terminus of the coding sequence contains an additional guanosine that is not present in the AaegL3.5 annotation (indicated in red). Altogether, these revisions result in an increased protein size from 470 to 785 amino acids. Of note, the sequence that was annotated by VectorBase as AAEL012437 translates into the RNA recognition motif (RRM) that is now part of the Veneno gene. Arrows 1-7 indicate the position and orientation of primers used to generate RT-PCR products used in (C), (E) and (F). Solid and dashes lines indicate the genome regions corresponding to the RNA sequencing data shown in (B) and (D), respectively. **(B)** RNA sequencing data from the indicated tissues corresponding to the genome stretch indicated by the solid line in (A) shows a similar level of expression of transcripts annotated as AAEL012437 and AAEL012441. **(C)** RT-PCR products amplified from Aag2 cDNA using the indicated primer combinations confirm that the sequences annotated as AAEL012437 and AAEL012441 are part of the same transcript. The sizes of the amplicons correspond with our revised annotation. The sequence was confirmed by Sanger sequencing. **(D)** RNA sequencing data corresponding to the region indicated by the dashed line in (A) show that the sequence annotated as intron 2 is part of the mature mRNA. **(E)** RT-PCR products amplified from Aag2 cDNA using the indicated primer combinations. The sizes of these products correspond to our revised annotation. The presence of the intron 2 sequence as part of the mature mRNA was also confirmed by Sanger sequencing. **(F)** Sanger sequencing of PCR-products generated from both Aag2 and Ae. aegypti genomic DNA using primers 6 and 7 reveal the presence of a non-annotated guanosine residue. This additional residue causes a frameshift with respect to the VectorBase annotation, extending the coding sequence at the carboxyl terminus. RNA sequencing data in (B) and (D) is taken from the genome browser for Ae. aegypti supercontig 1.697 (http://aedes.caltech.edu/) (1); numbers on top show nucleotide position on the scaffold and the AaegL3.5 annotation on VectorBase is depicted in blue.

# Supplemental Figure 5.

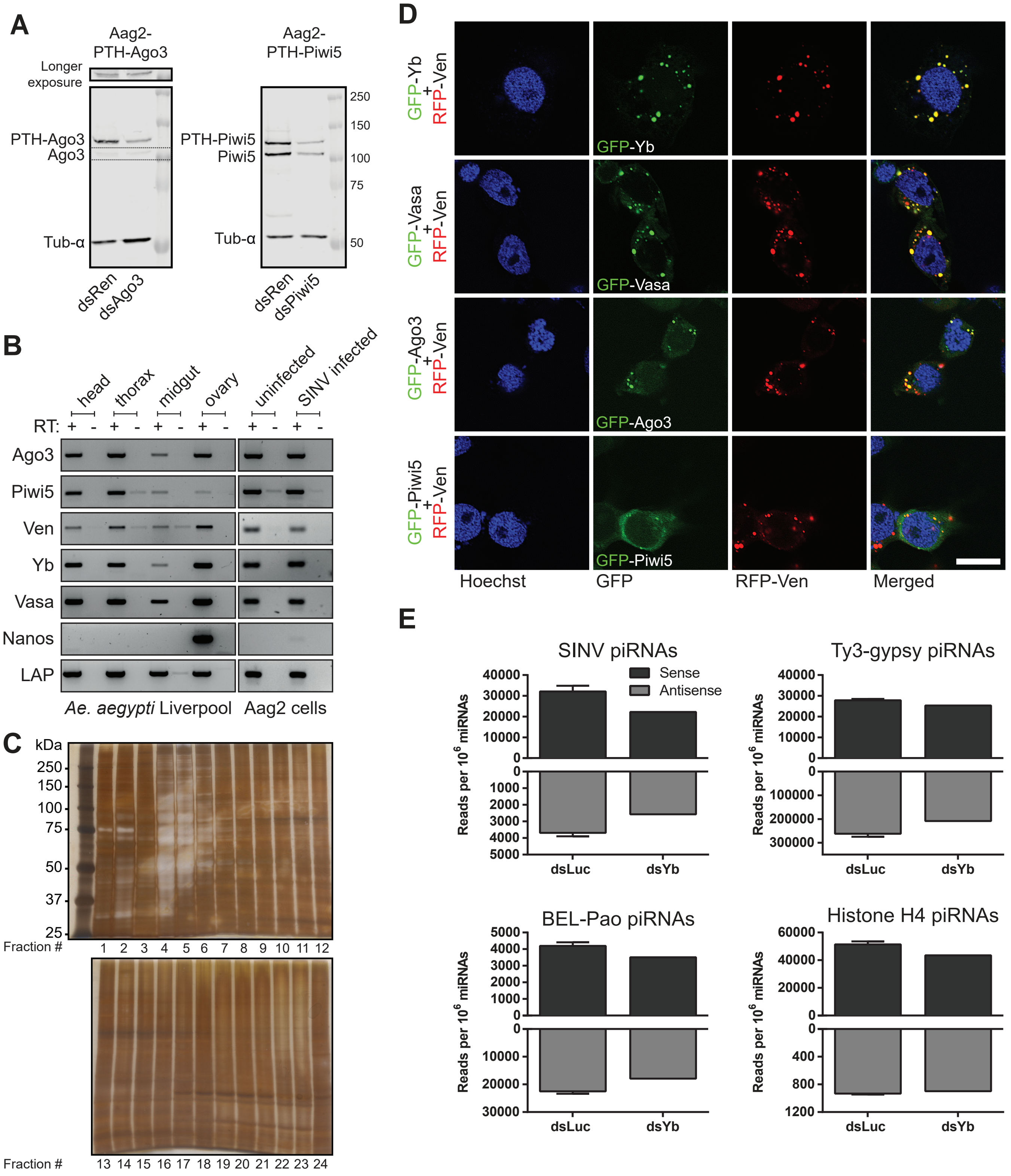


**Figure S5. Veneno associates with ping-pong partners Ago3 and Piwi5 and helicases Yb and Vasa in Ven-bodies**
**(A)** Antibodies were raised against Ae. aegypti Ago3 and Piwi5. Western blot of Ago3 (left) and Piwi5 (right) in Aag2 cell lines stably expressing protA-TEV-6xHis (PTH) tagged PIWI proteins. The samples were harvested at 48 hours post transfection of dsRNA against the indicated genes. dsRNA targeting Renilla Luciferase (dsRen) was used as control. Tubulin-α serves as a loading control. The dashed line indicates the area corresponding to the inset, depicting a longer exposure of the signal for endogenous Ago3, which is lowly expressed. **(B)** RT-PCR on indicated dissected mosquito tissues (left panel) and uninfected and SINV-infected Aag2 cells (right panel) with primers specific for Ago3, Piwi5, Ven, Yb, Vasa, Nanos, and LAP. Nanos serves as a germline-specific marker. These sequences were confirmed by Sanger sequencing. **(C)** Silver-stained SDS-PAGE gel loaded with fractions of a sucrose gradient of lysates from Aag2 cells stably expressing GFP-Ven. **(D)** Confocal images of Aag2 cells stably expressing GFP-tagged Yb, Vasa, Ago3 and Piwi5, transiently transfected with a vector encoding RFP-tagged Veneno. Scale bar represents 10µm. **(E)** The sum of normalized read counts of 25-30 nt piRNAs mapping to the Sindbis virus (SINV) genome, Ty3-gypsy transposons, BEL-Pao transposons, or Histone H4 mRNA upon knockdown of Yb (dsYb) and control knockdown (Firefly Luciferase, dsLuc). Read counts of three (dsLuc) and two (dsYb) independent libraries were normalized to the amount of miRNAs present in those libraries and analyzed separately for the sense (black) and antisense (grey) strands. Bars indicate mean +/- standard deviation.

## Supplemental Figure 6.

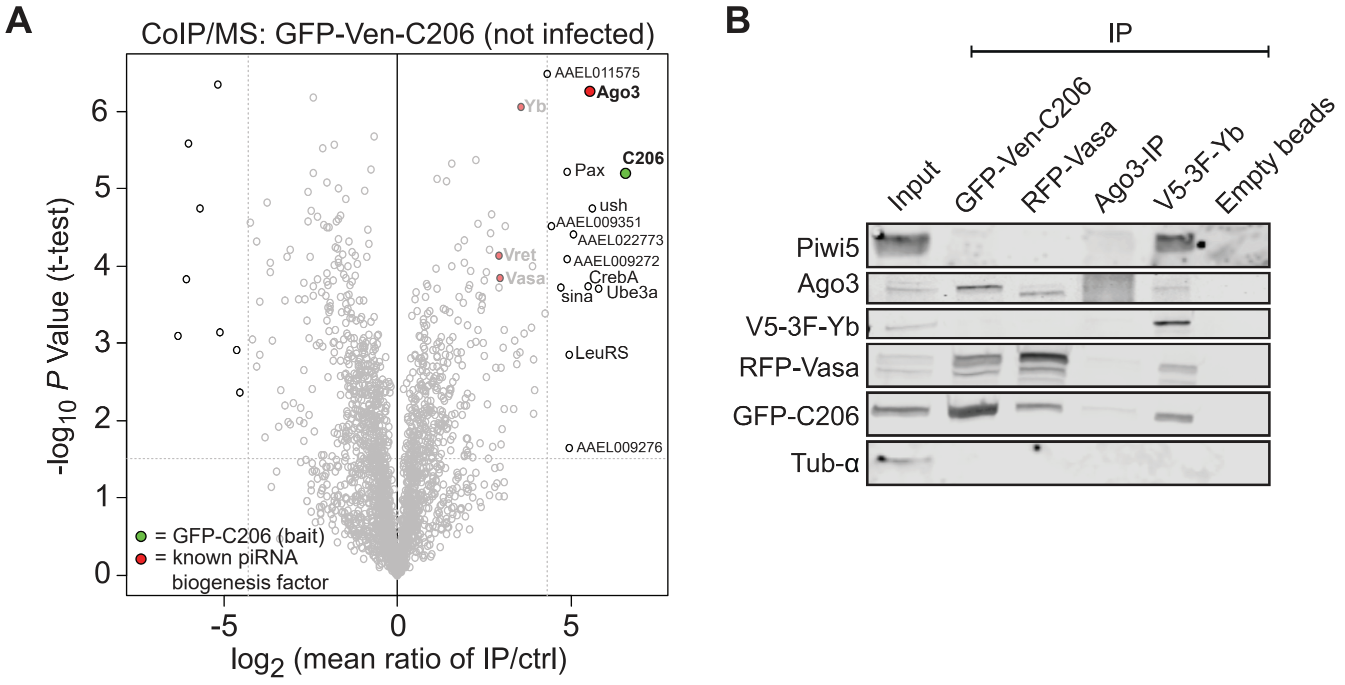


**C**

```
AaeVeneno  Tud_N   455 ----VIFAPFLGGYYRAKVLAV-----NGEKLQVFYVDFGNIDKVEWKESREIA--------------DEG 502
Dme CG9684 Tud_C   527 E---YVLARFEGSWYRGKVEQIIVVPRQQTKYRVMYLDYTNVEDITEM--------------------DIR 574
AaeVeneno  Tud_C   688 ----VCLVFHQDDWSRALYDIS-------D-GSYMLLDVGIITSVPAANVRRFPP--------------QL 732
DmeCG9684  Tud_N   189 ----LCLVHFNGMYVRAKMCKK-----LREVSHLFLVDLGIKHSGPFFDFKDINDELKALPCSSQLVQLKN 250
DmeTSN             753 GDLVAAQFTLDNQWYRAKVERV-----QGSNATVLYIDYGNKETLPT---------------------NRL 797
DmeTud11          2400 ----VAQFPEDEVFYRAQIRKVL----DDGKCEVHFIDFGNNAVT--QQFRQLPEELAKP--ARYSRHCEL 2458
                                    :  *.                      :*
```

**Figure S6. Association of Ven with Ago3, Yb and Vasa is independent of the N-terminal RRM.**
**(A)** Identification of proteins interacting with the truncated GFP-Ven transgene lacking the N-terminal RRM (Ven-C206) by label-free quantitative (LFQ) mass spectrometry. Statistical enrichment of proteins in the Ven-C206-IP was determined using a permutation-based FDR-corrected t-test. The LFQ-intensity of GFP-Ven-C206 IP over a control IP using the same lysate and non-specific beads (log2-transformed) is plotted against the -log10 P value. Interactors with an enrichment of log2 fold change > 4.3; -log10 P value > 1.5 are indicated. Proteins in the top right corner represent the bait protein in green (Ven) and its interactors. Orthologs of known piRNA biogenesis factors in D. melanogaster are indicated in red. Interactors of Ven-C206 were named after their closest Drosophila orthologue or the VectorBase GeneID. **(B)** Reciprocal immunoprecipitation of the GFP-tagged Ven-C206 transgene and RFP-tagged Vasa, V5-3xflag-tagged Yb and Ago3, using antibodies against GFP, RFP, V5 and Ago3, respectively. Membranes were probed with antibodies against GFP, RFP, flag, Ago3, Piwi5 and tubulin-α as indicated. **(C)** Multiple sequence alignment of TUDOR domains from Veneno, its Drosophila (Dme) orthologue CG9684, and Drosophila TSN and Tudor, of which crystal structures have been solved (2,3). Residues predicted to be involved in sDMA recognition sites are marked in yellow; asterisks (*) mark identical residues; colon (:) marks conserved substitutions; a period (.) marks a substitution by weakly similar residues. The second TUDOR domain of Veneno and the first TUDOR domain of CG9684 lack the aromatic cage residues required for sDMA recognition. T-Coffee (http://www.ebi.ac.uk/Tools/msa/tcoffee/) was used to align the sequences.

**Supplemental Figure 7.**

**A.** Related to Figure 5A

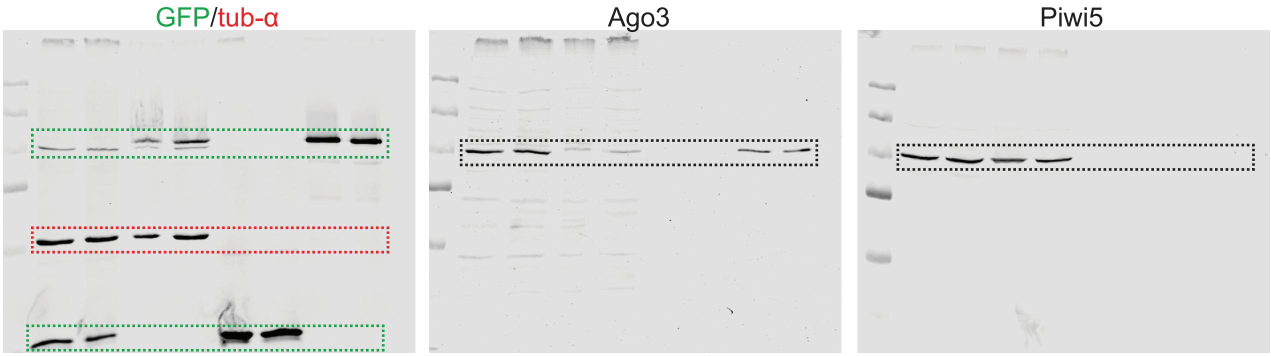


**B.** Related to Figure 5D

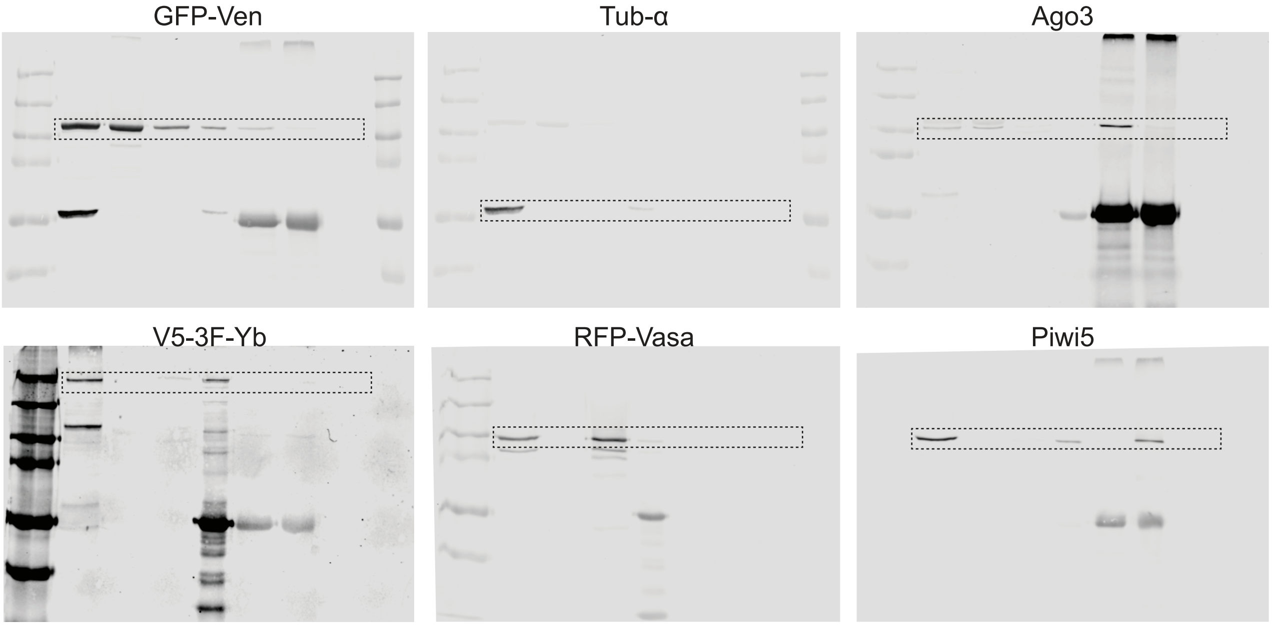


**C.** Related to Figure 5e

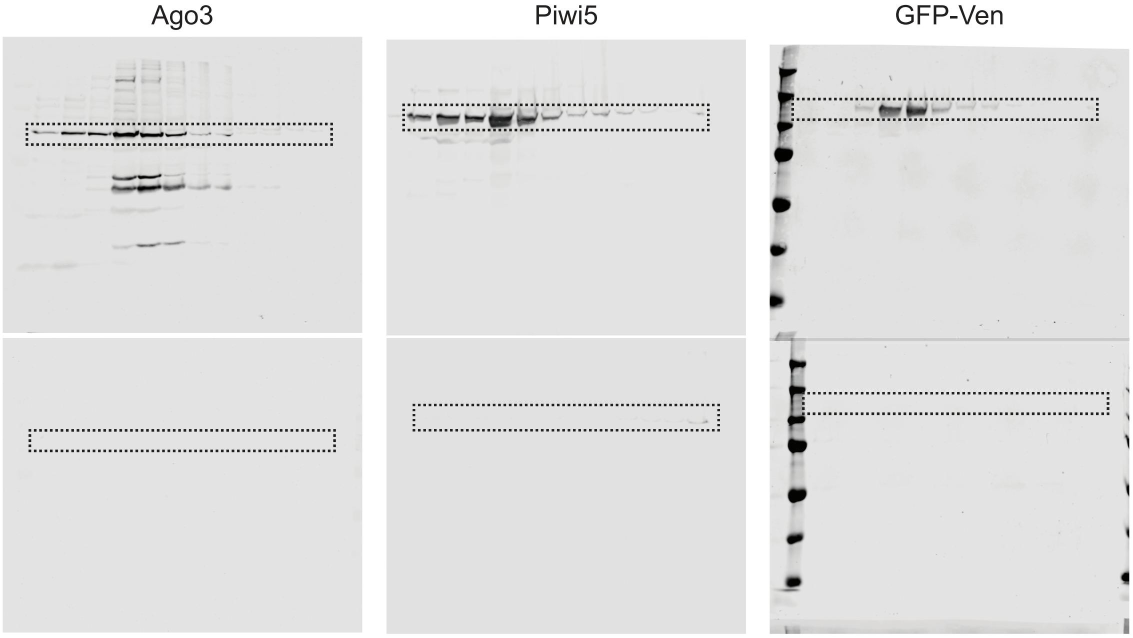

## **D.** Related to Figure 6C

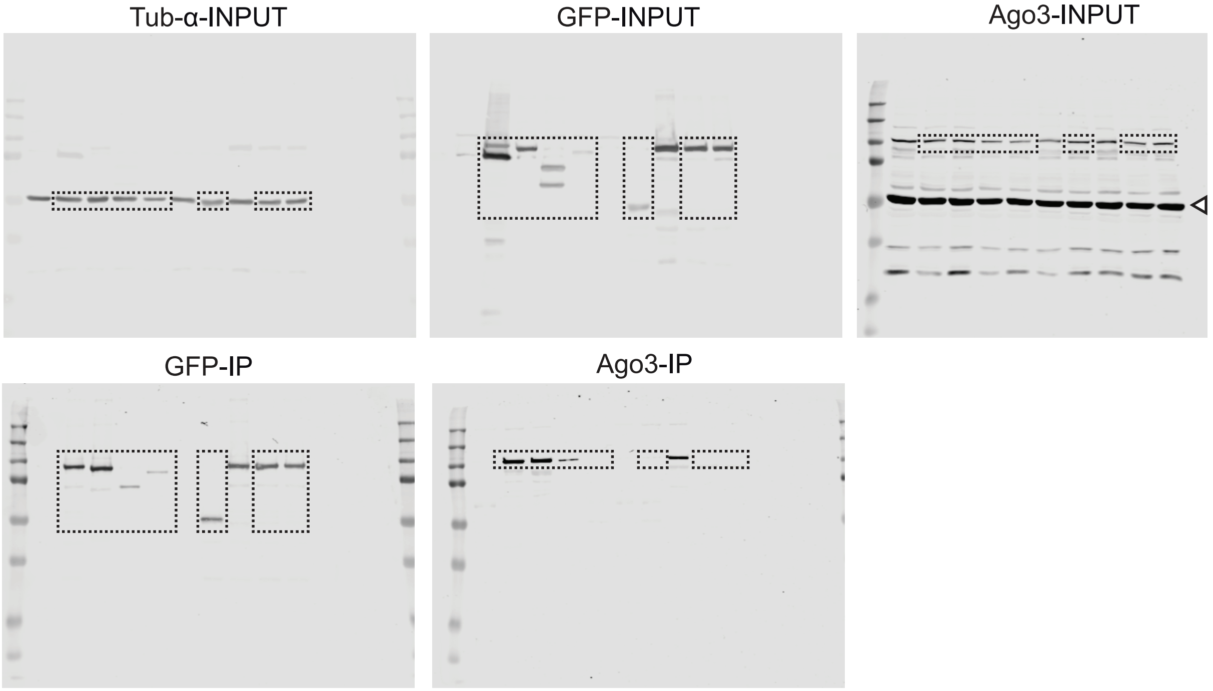


## **E.** Related to Figure 6D

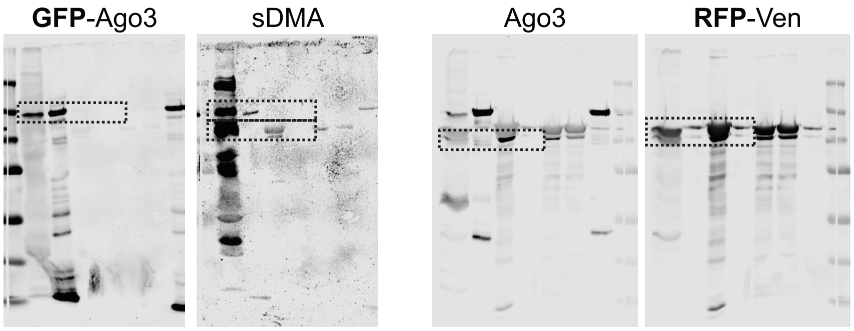


**Figure S7. Full gel images for Western Blots used in figures 5A, 5D, 5E, 6C and 6D.**
Dashed lines indicate the regions used for the figures in the main text. The arrowheads in the Ago3-Input blot in (D) indicates signal from the tubulin-α antibody.

## Supplemental Table 1.

| | ABBREVIATION | *DME.* ORTHOLOGUE | NCBI ID | VECTORBASE ID | PEPTIDES | DOMAINS |
|---|---|---|---|---|---|---|
| **BOTH IPS** | Ven | CG9684 | ref\|XP_001662584.2\| | AAEL012437 | 82 | Zf-MYND, RRM, TUDOR (x2) |
| | Ago3 | Ago3 | ref\|XP_001652945.1\| | AAEL007823 | 32 | PIWI, PAZ, L1 |
| | RecQ5 | RecQ5 | ref\|XP_021705880.1\| | AAEL018344 | 6 | RecQ, MDN1, DUF1777 |
| | RpL39 | RpL39 | ref\|XP_021708642.1\| | AAEL020290 | 2 | L39 |
| | stumps | stumps | ref\|XP_021707804.1\| | AAEL021877 | 13 | DBB, SoxR, ANK |
| | Yb | Yb | ref\|XP_021705665.1\| | AAEL001939 | 18 | DEXDc-helicase, TUDOR (x3) |
| **MOCK-INFECTED IP** | AAEL018022 | CG2061 | ref\|XP_021700942.1\| | AAEL018022 | 8 | LANCL |
| | His2Av | His2Av | ref\|XP_021693196.1\| | AAEL023603 | 6 | H2A |
| | RpL36A | RpL36A | ref\|XP_001648064.1\| | AAEL003942 | 7 | L44 |
| | Sesn | Sesn | ref\|XP_021697010.1\| | AAEL002487 | 4 | PA26 |
| | Rab39 | Rab39 | ref\|XP_001653610.1\| | AAEL008959 | 6 | Rab39 |
| **SINV-INFECTED IP** | Rheb | Rheb | ref\|XP_021711486.1\| | AAEL008179 | 5 | RheB |
| | RpS29 | RpS29 | ref\|XP_001652933.1\| | AAEL007824 | 2 | S14p/S29e |
| | Mocs1 | Mocs1 | ref\|XP_021697606.1\| | AAEL000126 | 4 | SkfB, moaC |
| | Rho1 | Rho1 | ref\|XP_001651046.2\| | AAEL018059 | 5 | STKc_ROCK, PH_ROCK, SbcC, C1, SMC_N |
| | AAEL006227 | CG4611 | ref\|XP_001657591.2\| | AAEL006227 | 2 | PPR (x6) |
| | nonA | nonA | ref\|XP_021705281.1\| | AAEL017116 | 28 | RRM (x2), NONA, DUF3552 |
| | vret | vret | ref\|XP_021695080.1\| | AAEL020359 | 11 | Zf-MYND, RRM, TUDOR (x2) |
| | mRpL24 | mRpL24 | ref\|XP_001655972.2\| | AAEL012264 | 6 | L24 |
| | RpL21 | RpL21 | ref\|XP_001652846.1\| | AAEL007715 | 10 | L21e |

**Table S1. Factors identified by Ven-interactome mass spectrometry**
A $\log_2$ fold change > 4.3 accompanied by a $-\log_{10}$ *P* value > 1.5 was used as cutoff.

# Supplemental Table 2.

| ABBREVIATION | *DME.* ORTHOLOGUE | NCBI ID | VECTORBASE ID | PEPTIDES | DOMAINS |
|---|---|---|---|---|---|
| Ven | CG9684 | ref\|XP_001662584.2\| | AAEL012437 | 93 | Zf-MYND, RRM, TUDOR (x2) |
| Ago3 | Ago3 | ref\|XP_001652945.1\| | AAEL007823 | 95 | PIWI, PAZ, L1 |
| AAEL011575 | CG32486 | ref\|XP_001661753.2\| | AAEL011575 | 24 | RING, Sina |
| Pax | Pax | ref\|XP_021702270.1\| | AAEL019826 | 29 | LIM (x4) |
| ush | ush | ref\|XP_021703888.1\| | AAEL020615 | 21 | Zf-C2H2 (x2) |
| AAEL009351 | CG8993 | ref\|XP_021712657.1\| | AAEL021624 | 2 | TRX |
| AAEL022773 | - | ref\|XP_021700284.1\| | AAEL022773 | 6 | TUDOR |
| AAEL009272 | CG3587 | ref\|XP_021705202.1\| | AAEL009272 | 13 | KTI12 |
| CrebA | CrebA | ref\|XP_021709690.1\| | AAEL003646 | 2 | CREB3 |
| Ube3a | Ube3a | ref\|XP_021710239.1\| | AAEL012500 | 22 | HECTc, AZUL |
| sina | sina | ref\|XP_001653865.1\| | AAEL009614 | 5 | RING, Sina |
| LeuRS | LeuRS | ref\|XP_001657794.2\| | AAEL006415 | 39 | - |
| AAEL009276 | CG11596 | ref\|XP_001653776.1\| | AAEL009276 | 10 | N2227 |

**Table S2. Factors identified by Ven-C206-interactome mass spectrometry**
A $\log_2$ fold change > 4.3 accompanied by a $-\log_{10}$ *P* value > 1.5 was used as cutoff.

## SUPPLEMENTAL MATERIALS AND METHODS

### Detection of TUDOR orthologs from *Drosophila* and *Ae. aegypti*

The *Drosophila* proteome was scanned using the conserved TUDOR multi-domain sequence (pfam00567: LPEGSTIDVVVSHIESPSHFYIQPVSDSKKLEKLTEELQEYYASKPPESLPPAVGDGCVAAFSE DGKWYRARVTESLDDGLVEVLFIDYGNTETVPLSDLRPLPPEFESLPPQAIKCSLAG) in HHpred 2.18 (cutoff E ≤ 0.01) (4). Homologous sequences were subsequently used as input for iterative searches using Jackhmmer 2.7 to predict all *D. melanogaster* and *Ae. Aegypti* TUDOR domains (5). We aligned identified TUDOR-domain sequences using T-Coffee to generate a neighbor joining tree based on sequence identity without correcting for multiple substitutions (6). For Tudor genes that contain multiple TUDOR domains (e.g. AAEL007841), the phylogenetic tree was used to guide identification of the domains that had the highest degree of similarity with a *D. melanogaster* TUDOR domain. Using only these TUDOR domain sequences, a new neighbor joining tree was generated with T-coffee, which is shown in Figure 1. A combination of SMART-, HHPred-, Pfam- and Hmmscan-mediated domain prediction as well as the available literature was used to determine protein domain composition (5,7,8). The prion-like amino acid composition (PLAAC) web application (http://plaac.wi.mit.edu/) was used to scan the Ven protein sequence for sequences with low amino acid complexity (9).

### Cells and viruses

Aag2 cells were cultured at 25°C in Leibovitz's L-15 medium (Invitrogen) supplemented with 10% fetal bovine serum (Gibco), 50 U/ml Penicillin, 50 µg/mL Streptomycin (Invitrogen), 1x Non-essential Amino Acids (Invitrogen) and 2% Tryptose phosphate broth solution (Sigma). The viruses used in this study are recombinant Sindbis viruses that contain a second subgenomic promoter located downstream of the structural genes from which GFP (SINV-GFP) is expressed when indicated. Viruses were produced in BHK-21 cells as previously described (10).

### Stable cell lines

We generated plasmids containing a polyubiquitin promoter driving the expressing of GFP-tagged proteins linked to a puromycin resistance gene via a T2A polyprotein self-cleavage site. Approximately $3 \times 10^6$ Aag2 cells were transfected with 5µg of plasmid. Three hours post-transfection, medium was refreshed and 48 hours later it was replaced with medium containing puromycin (2 µg/mL) as a selection marker. The medium was replaced every 3-4 days, and cells were kept under puromycin pressure throughout. All cell lines were polyclonal.

### Generation of plasmids

Wildtype and mutant Veneno as well as Vasa (AAEL004978) sequences were sub-cloned into the pAGW, pARW (Carnegie Gateway vector collection) or pUGW expression vectors, using Gateway cloning (Invitrogen). The pUGW expression vector was derived from the pUbB-GW vector (kindly provided by Gorben Pijlman, University of Wageningen), which was generated by exchanging the OpIE2 promoter from pIB-GW (Thermo Scientific) by the poly-ubiquitin promoter from pGL3-Pub (11). A PCR product of pPUb was created with BspHI and SacI sites, which was then ligated into the BspHI and SacI-digested pIB-GW vector. To generate the pUGW vector, the GFP sequence for N-terminal tagging of proteins was ligated into the pPUbB-GW vector using the SacI restriction sites. AttB1 and AttB2 recombination sites flanking indicated Ven- and Vasa-sequences were added during PCR amplification from Aag2 complementary DNA (cDNA). Donor vectors were generated through BP-recombination of the produced PCR products with pDONR/Zeo (Invitrogen). Subsequently, gene fragments were cloned into the expression vectors by LR-recombination (Invitrogen). Point mutations in Ven-expression plasmids were introduced by site-directed mutagenesis using In-fusion HD Cloning (Takara). V5-3xflag tagged Yb (AAEL001939) was cloned into an expression vector based on the pAc5.1 (Invitrogen) backbone using In-fusion.

The following primers were used for cloning (**boldcase** refers to restriction sites, lowercase to AttB1/AttB2 recombination sites, and additional stopcodons are shown in *italic*):

| | |
|---|---|
| FW-SacI-EGFP | CCACC**GAGCTC**ATGGTGAGCAAGGGCG |
| RV-SacI-EGFP | CGGTG**GAGCTC**CCTTGTACAGCTCGTCCATGC |
| FW-AttB1-Ven_N | ggggacaagtttgtacaaaaaagcaggcttcATGCCTCGTAACGATTCC |
| RV-AttB2-Ven_N | tggtgaccactttgtacaagaaagctgggtt*ttattatta*GGCATCCACGAGAGAAATGGA |
| FW-AttB1-C91_N | ggggacaagtttgtacaaaaaagcaggcttcAAAGCCGTTGATCCGGCC |
| FW-AttB1-C199_N | ggggacaagtttgtacaaaaaagcaggcttcTGCTGGTCTTGCACCAAGATG |
| FW-AttB1-C206_N | ggggacaagtttgtacaaaaaagcaggcttcCCTAGCTATGAGTGCCGCTGC |
| FW-AttB1-C234_N | ggggacaagtttgtacaaaaaagcaggcttcATGCCTCGACTGGTACCCATT |
| FW-AttB1-C400_N | ggggacaagtttgtacaaaaaagcaggcttcGGTGCAATGGTCAAGATTACCG |
| RV-AttB2-N670_N | ggggaccactttgtacaagaaagctgggt*ttattatta*GCTACAATGCTCCAGGATGGC |
| RV-AttB2-N399_N | ggggaccactttgtacaagaaagctgggt*tttattatta*TTCCCGAGGGAACGGTCC |
| FW-G463A/Y465A | GGCGCATACGCCCGGGCCAAAGTTCTGGCGGTGAA |
| RV-G463A/Y465A | CCGGGCGTATGCGCCGAGGAAGGGAGCAAAGATGA |
| FW-D483A/N486A | TCGCCTTTGGCGCCATCGATAAGGTCGAGTGGAAG |
| RV-D483A/N486A | TGGCGCCAAAGGCGACGTAGAACACCTGCAACTTC |
| FW-Yb/AAEL001939_Inf | gataagctttctaga**CTCGAG**ATGTTCGAAGACGACACCAT |
| RV-Yb/AAEL001939_Inf | gagctcgcggccgct**CTCGAG**TTAACCATCCCGCAGGAAATA |
| FW-AttB1-Vasa/AAEL004978 | ggggacaagtttgtacaaaaaagcaggcttcTGCGATGAATGGGAGGATAATGA |
| RV-AttB2-Vasa/AAEL004978 | ggggaccactttgtacaagaaagctgggtt*ttattatta*ATCCCAGTCTTCTTCTGGTTC |

**dsRNA production**

PCR products flanked on both sites by the T7 promoter sequence were *in vitro* transcribed by T7 polymerase to produce dsRNA. The T7 sequence was either directly coupled to the primers used to generate these PCR products or introduced in a second PCR using universal primers that hybridize to a short GC-rich universal tag (UT) that was introduced in an initial PCR, as described previously (12). The following primers were used for dsRNA production (5’ to 3’ orientation, lowercase refers to the T7 promoter sequence, **bold** font to the universal tag):

| | |
|---|---|
| Fw-T7-Luc | taatacgactcactatagggagaTATGAAGAGATACGCCCTGGTT |
| Rv-T7-Luc | taatacgactcactatagggagaTAAAACCGGGAGGTAGATGAGA |
| Fw-T7-Piwi5 | taatacgactcactatagggagaGCCATACATCGGGTCAAAAT |
| Rv-T7-Piwi5 | taatacgactcactatagggagaCTCTCCACCGAAGGATTGAA |
| Fw-T7-AAEL021145 | taatacgactcactatagggagaGCTACCAGAGCCAGAGCAAC |
| Rv-T7-AAEL021145 | taatacgactcactatagggagaTCGGTCAACGCGTAATCATA |
| Fw-T7-AAEL027589 | taatacgactcactatagggagaTCGGATGCGTATCATTACGA |
| Rv-T7- AAEL027589 | taatacgactcactatagggagaAATTCCTTCGTGCTGTTTGG |
| Fw-UT-AAEL000293 | **gcccgacgc**TCTATTCCGAACGGCCGC |
| Rv-UT-AAEL000293 | **cgcctcggc**CAGCCGTCGTGTCTGGTT |
| Fw-UT-Yb/AAEL001939-1 | **gcccgacgc**AGCAAACCAGCCTTAAATAATCA |
| Rv-UT-Yb/AAEL001939-1 | **cgcctcggc**GGCTGGAATTGGGTCTTCAA |
| Fw-UT-Yb/AAEL001939-2 | **gcccgacgc**CGATGTCCTACCAATAACGC |
| Rv-UT-Yb/AAEL001939-2 | **cgcctcggc**CTCTTGTCCCATGTTCCG |
| Fw-UT-Yb/AAEL001939-3 | **gcccgacgc**ATTGGAGCATACCTAGAGGC |
| Rv-UT-Yb/AAEL001939-3 | **cgcctcggc**ACCAATCATTGTAGTGTACGG |
| Fw-UT-Yb/AAEL001939-4 | **gcccgacgc**CGGCATTTGTTGGACCCG |
| Rv-UT-Yb/AAEL001939-4 | **cgcctcggc**GACAGTCCACGCACCTCA |
| Fw-UT-AAEL002036 | **gcccgacgc**GCCCTGCCGGATGAGTAC |
| Rv-UT-AAEL002036 | **cgcctcggc**CGTCGTCCAAGGCCACAA |
| Fw-UT-AAEL004290 | **gcccgacgc**GCTCACAGAGGAAGCGGG |
| Rv-UT-AAEL004290 | **cgcctcggc**TATGGCAGGGCTAGGAGC |
| Fw-UT-AAEL008101 | **gcccgacgc**TGTGCTTAGCGAGGCGAC |
| Rv-UT-AAEL008101 | **cgcctcggc**CCAGCGGTGGCAGATTCT |
| Fw-UT-AAEL008101-RB | **gcccgacgc**GCCACATTCTACATGATCG |
| Rv-UT-AAEL008101-RB | **cgcctcggc**ACGTGGAGGAGATTGTCC |
| Fw-UT-AAEL008101-RC | **gcccgacgc**CAGAGCATGAGCAGTGC |
| Rv-UT-AAEL008101-RC | **cgcctcggc**TGGCGTGGAGAATGG |
| Fw-T7-Vasa/AAEL004978-1 | taatacgactcactatagggagaAATGATGCAACCGGAAAATC |
| Rv-T7-Vasa/AAEL004978-1 | taatacgactcactatagggagaCTCCGTTTTCACCTGGTCAT |
| Fw-UT-Vasa/AAEL004978-2 | **gcccgacgc**GAGGCGACAACGAAGG |

| | |
|---|---|
| Rv-UT-Vasa/AAEL004978-2 | **cgcctcggc**CAGGCCATTAAATCTCGC |
| Fw-UT-AAEL008112 | **gcccgacgc**TCCACAACGGGGGCATTC |
| Rv-UT-AAEL008112 | **cgcctcggc**CTTGTGTAGGGCAGGGGC |
| Fw-UT-AAEL008431 | **gcccgacgc**TGGACGAAAAGCCGGCTT |
| Rv-UT-AAEL008431 | **cgcctcggc**CAGGTAGCTGTGGCGCTT |
| Fw-UT-AAEL008700 | **gcccgacgc**AACAGACGGTGGCCATCG |
| Rv-UT-AAEL008700 | **cgcctcggc**TCTAGGACGGTCGGGCTC |
| Fw-UTAAEL022773 | **gcccgacgc**GCGGATACAGCTGCCCAA |
| Rv-UT-AAEL022773 | **cgcctcggc**GGACGGCTTGACACACCA |
| Fw-UT-AAEL020359 | **gcccgacgc**CAGCCGGAATCAGCGTCA |
| Rv-UT- AAEL020359 | **cgcctcggc**TGTCGTCTAGTCGGGCCA |
| Fw-UT-AAEL009416-1 | **gcccgacgc**AGCACCTCTGCAGCAGTG |
| Rv-UT-AAEL009416-1 | **cgcctcggc**AGGTATGAGGCAACGCGG |
| Fw-UT-AAEL009416-2 | **gcccgacgc**CAGGAAGATGATGATGTAAAGG |
| Rv-UT-AAEL009416-2 | **cgcctcggc**TGCTTTTGCCGTTCTTATC |
| Fw-UT-AAEL009987/023919 | **gcccgacgc**GAGCGCGACCGGTATCAA |
| Rv-UT-AAEL009987/023919 | **cgcctcggc**GGTTTTTCCACACAGGCCA |
| Fw-UT-AAEL010311 | **gcccgacgc**CGCATACAGTGTCGCGTG |
| Rv-UT-AAEL010311 | **cgcctcggc**GCCGTCATGCACTTTGCC |
| Fw-UT-Ven | **gcccgacgc**ACGAAACGCAACACCATTG |
| Rv-UT-Ven | **cgcctcggc**TCATGGCGTTTTCCGGGA |
| Fw-UT-AAEL012276 | **gcccgacgc**AGCAGCTCTTCTGACGGA |
| Rv-UT-AAEL012276 | **cgcctcggc**TGATTGGGTCGCATGCGT |
| Fw-UT-AAEL013072 | **gcccgacgc**TCGGGCTGAAGTGATCGC |
| Rv-UT-AAEL013072 | **cgcctcggc**CCTTGGCATGACCCTCGG |
| Fw-UT-AAEL023716 | **gcccgacgc**CTGCCATGTCCATCGCGA |
| Rv-UT-AAEL023716 | **cgcctcggc**ATCGCAAAGTCCAGCCGG |
| F-T7-universal primer | taatacgactcactatagggaga**gcccgacgc** |
| R-T7-universal primer | taatacgactcactatagggaga**cgcctcggc** |

**Transfection of plasmids and dsRNA**

For immunoprecipitation and immunofluorescence analyses, Aag2 cells were transfected with expression vectors using X-tremeGENE HP (Roche) according to the manufacturer's instructions. For knockdown experiments, dsRNA was transfected into Aag2 cells using X-tremeGENE HP and transfected again 48 hours after the initial transfection to increase knockdown efficiency. Three hours after each transfection, medium was refreshed with supplemented Leibovitz's medium and infected with SINV where indicated. Samples were harvested 48 hours post infection.

**RNA isolation**

Total RNA was isolated using Isol-RNA Lysis reagent (5 PRIME) or RNA-solv reagent (Omega) following the manufacturer's instructions. Briefly, 200 µl of chloroform was added to 1 ml of lysate. After harsh mixing and centrifugation, the aqueous phase was collected and RNA was precipitated by adding one volume of isopropanol. RNA pellets were washed 2-3 times in 85 % ethanol and dissolved in nuclease-free water.

**Small RNA Northern Blotting**

3-5 µg of total RNA was diluted in 2x loading buffer (93.65% formamide, 18mM EDTA, 0.025% SDS, 0.025% Xylene cyanol, 0.025% Bromophenol Blue) and size separated on 7M urea 15 % polyacrylamide 0.5x TBE gels by gel-electrophoresis. RNA was then transferred to Hybond Nx nylon membranes (Amersham) and cross-linked using 1-ethyl-3-(3-dimethylaminopropyl)carbodiimide hydrochloride as described in (13). The membranes were pre-hybridized in Ultrahyb Oligo hybridization buffer (Ambion) for at least 30 min at 42°C under constant rotation. $^{32}$P end-labelled DNA oligonucleotides were added directly into the buffer and allowed to hybridize overnight at 42°C under constant rotation. Afterwards, membranes were washed three times in wash buffers containing SDS and decreasing concentrations of SSC as described in(12). Probes used for northern blotting were:

| | |
|---|---|
| SINV-7903 (+) | GGTTGCTTCTTCTTCTTCTCCTGCGTTT |
| SINV-7940 (+) | AGTGCCATGCGCTGTCTCTTTCCGGGTTTG |

| | |
|---|---|
| SINV-7969 (+) | TCGAACAATCTGTCGGCCTCCAACTTAA |
| SINV-8040 (+) | GCAGAGGTTTCATTACCTTTCCTTCCAT |
| miR-2940 3p | AGTGATTTATCTCCCTGTCGAC |
| H4-149 (+) | GCACTCCACGGGTTTCCTCGTAGATAA |
| H4-183 (+) | AGCATCACGAATGACATTTTCCAGGAAT |
| H4-218 (+) | ACGGTTTTACGCTTGGCGTGTTCAGTGT |
| H4-257 (+) | CCCTGACGCTTCAGAGCGTAGACAACAT |
| PCLV-S-412 (+) | AGTCCTTGGTATTCAAACTCAGCCAATG |
| PCLV-S-476 (+) | GGTCTTGATCGGCCAGCTGCGTTTCCC |
| PCLV-S-571 (+) | CTTCCCTGTTTCATTAGTTCGAGTTAGCA |
| U6 snRNA | GATTTTGCGTGTCATCCTTGTGCAGGGGCCATGCTAA |

**Reverse transcription and (quantitative) PCR**

Total RNA was DNaseI treated (Ambion) and reverse transcribed using Taqman reverse transcriptase (Life Technologies) and SYBR-green qPCR was performed using the GoTaq qPCR system (Promega) according to recommendations of the manufacturers. Expression levels of target genes were internally normalized against expression of the housekeeping gene lysosomal aspartic protease (LAP) and fold changes were calculated using the 2^(-ΔΔCT) method (14). RT-PCR to produce Ven-amplicons from genomic or cDNA used for Sanger sequencing was performed using proof-reading Phusion polymerase (NEB). The following primers were used for (q)RT-PCR:

| | |
|---|---|
| qFw-SINV-NSP4 | AACTCTGCCACAGATCAGCC |
| qRv-SINV-NSP4 | GGGGCAGAAGGTTGCAGTAT |
| qFw-SINV-Capsid | CTGGCCATGGAAGGAAAGGT |
| qRv-SINV-Capsid | CCACTATACTGCACCGCTCC |
| qFw-LAP | GTGCTCATTCACCAACATCG |
| qRv-LAP | AACTTGGCCGCAACAAATAC |
| qFw-Piwi5 | ACGGCATCACATCGAGACTC |
| qRv-Piwi5 | CGACCTCCACGCTGTCCTC |
| qFw-Ago3 | CTCCAGACGACGGTTTTGGA |
| qRv-Ago3 | GCAGGTACGAAATTGGCTGC |
| qFw-AAEL000293 | ACAAGAAGGACCGCAGACTG |
| qRv-AAEL000293 | TCGATTAGTTGGTGGCCGAG |
| qFw-Yb/AAEL001939 | GTTGCCGGATTGTCAGCATC |
| qRv-Yb/AAEL001939 | GGCAATCGGCGGAATTCTTC |
| qFw-AAEL002036 | GCCTGGAGGTGTACTGTTCC |
| qRv-AAEL002036 | ATTCGACTTGAGGCCTGCTC |
| qFw-AAEL004290 | CGATGATTCACTGCTTGGCG |
| qRv-AAEL004290 | ATCGTCCTCGCAGTCACATC |
| qFw-AAEL021145 | CGTCGTCGAAGAAATCCAAT |
| qRv-AAEL021145 | TGAACTTGCTCTGCAGGATG |
| qFw-AAEL008101 | TTCCAGGCCGTCCACTGT |
| qRv-AAEL008101 | GGATGTCTAGCACTTCGACG |
| qFw-AAEL008101-RB | AATAAACCCAAGGAAGAAGC |
| qRv-AAEL008101-RB | CGGTACAACAGATGTTAGGTATC |
| qFw-AAEL008101-RC | GGTGCCTGCTTAGCATAAG |
| qRv-AAEL008101-RC | GACGAGACGACACATCG |
| qFw-AAEL008112 | AGGTTCGGTAACATGCCCAG |
| qRv-AAEL008112 | TCTGGAAAACACGGACCACC |
| qFw-AAEL008431 | ACTCCTGAAGCATCGGAAGC |
| qRv-AAEL008431 | TATTCCTCCCACACTGCCAG |
| qFw-AAEL008700 | TTGTAGCAAGGCGTCCAACT |
| qRv-AAEL008700 | GATCCAATTCCGCCGGTTTG |
| qFw-AAEL022773 | AAACTGTCTGGATGTGGTTCTG |
| qRv-AAEL022773 | AAATGATTTCGTACGCTCGCG |
| qFw-AAEL020359 | CTCCGTTCTATGCGAGCAGA |
| qRv-AAEL020359 | CCGTTGATTTGGCCTTTGGG |
| qFw-AAEL009416 | AAAGAAGTGGGAGAGCAGCC |
| qRv-AAEL009416 | TTGACATTCGGCCGGATCAA |

| | |
|---|---|
| qFw-AAEL009987/023919 | GCTTGATCGAGCTGCAAGTC |
| qRv-AAEL009987/023919 | GTGCCCGGTACCATAGATCG |
| qFw-AAEL010311 | CGTTCCGAAAGACAGCGTTG |
| qRv-AAEL010311 | GCAGGTAGGATTCGCAGTGA |
| qFw-AAEL012276 | GGTGGTGAAGTCAGTGTCGT |
| qRv-AAEL012276 | TCTTCCAGCTCCTTGAAGCG |
| qFw-Ven | TGGGCATCATTACCAGCGTT |
| qRv-Ven | TCGATCAACGCTCCGTGAAT |
| qFw-AAEL013072 | TTGAGCAGCGTTGAAAACCG |
| qRv-AAEL013072 | GGCTGGGATGCTGACTCATC |
| qFw-AAEL023716 | GTGGTCGCGATCCCTGTAAT |
| qRv-AAEL023716 | TTGCTACCCAGGAACGTCAC |
| qFw-AAEL027589 | CGGGTTGCTTATTCTCCTCA |
| qRv-AAEL027589 | GCCAAGAATTGTTTCGCAAT |
| qFW-Vasa/AAEL004978 | ACGCTGATATGGTCCCTGGA |
| qRV-Vasa/AAEL004978 | CATCTGATGACGGGTAGCGG |
| qFW-Nanos | GGGCGCATTTCAATCGTTCA |
| qRV-Nanos | GTCCAGTGGCCTGACAGTAC |
| 1-Fw-AAEL012437UTR | CAGCCGAATTGTGTGC |
| 2-Fw-AAEL012437ex1 | ATGCCTCGTAACGATTCC |
| 3-Fw-AAEL012441ex1 | GCGGTGCCTTCTACTGC |
| 4-Rv-AAEL012441in2 | TTGTCTTTGTTGTTGCTGC |
| 5-Rv-AAEL012441ex3 | TCTGACCGCGTTGACG |
| 6-Fw-AAEL012441ex4 | ACTCTAACGTCTACAAACCGG |
| 7-Rv-AAEL012441in4 | AGTTATACAATCAAGCCAAACAC |

**Analyses of small RNA deep sequencing libraries**

Deep sequencing libraries were sequenced on an Illumina Hiseq4000 machine by Plateforme GenomEast (Strasbourg, France). Image analysis and base calling were performed using RTA 2.7.3 and bcl2fastq. Initial quality control was performed using FastQC. Subsequent manipulations were performed in Galaxy (15). First, the 3' adapters were clipped from the small RNA sequence reads using the FASTX Clip adapter software. Reads were then mapped to the SINV-pTE3'2J-GFP virus genome, transposable elements sequences available on TEfam (http://tefam.biochem.vt.edu; downloaded 13/06/2017) or *Ae. aegypti* histone H4 sequences using Bowtie allowing one mismatch (16). Small RNA libraries were normalized against the number of reads that map to published pre-microRNAs sequences deposited in miRBase v.21 without allowing mismatches. Similar results have been obtained using the library size for normalization. Size profiles of indicated small RNA populations were generated and the counts of siRNAs (21nt reads) or piRNAs (25-30nt reads) derived from the sense and antisense strands of indicated RNA substrates were plotted. Sequence logos were generated using the WebLogo3 tool available on the Galaxy webserver (17,18). The overlap probability of viral piRNAs has been determined using the small RNA signature tool available at the Mississippi Galaxy instance (mississippi.fr). Sequence data have been deposited in the NCBI sequence read archive under SRA accession SRP127210.

**Fluorescent imaging and analyses**

To evaluate the subcellular localization of Ven transgenes, Aag2 cells expressing GFP-tagged constructs were fixed on coverslips using 4% paraformaldehyde. After permeabilization with 0.1% Triton X-100 in PBS, nuclei were stained using Hoechst reagent, washed and mounted onto microscope slides using Mowiol. Confocal images were taken using the Olympus FV1000 microscope after which a Gaussian filter was applied for smoothing and edge detection in FIJI (19). Cells used for quantification of cytoplasmic GFP-signal intensity were imaged using the Zeiss Axio Imager Z1 equipped with ApoTome technology for increased axial resolution. The cytoplasm of 46-56 cells expressing indicated transgenes were traced, and the coefficient of variation (Cv) was calculated from the mean intensity value (μ) and standard deviation (σ) as a measure for signal granularity as below using FIJI(19).

$$Cv = \sigma/\mu$$

**Generation of Ago3 and Piwi5 antibodies**

Anti-Ago3 antibodies were raised against a mix of two selected peptides (**TSGADSSESDDKQSS** and IIYKRKQRMSENIQF) by immunization of two rabbits (both rabbits received both peptides) (Eurogentec). After an initial immunization and 3 boost immunizations at t = 14 days, t = 28 days and t = 56 days, final bleeds were collected at t = 87 days and pooled. Antibodies were purified against each peptide separately and specificity was determined by Western blot analysis (Figure S6A). Anti-Piwi4, -Piwi5, and Piwi6 antibodies were obtained using the same protocol (Piwi4: **CHEGRGSPSSRPAYSS** and CHHRESSAGGRERSGN; Piwi5: **DIVRSRPLDSKVVKQ** and CANQGGNWRDNYKRAI; Piwi6: **MADNPQEGSSGGRIRC** and CRGDHRQKPYDRPEQS). Antibodies purified against the peptides in **bold** font were used throughout this study.

**Immunoprecipitation and western blotting**

Aag2 cells expressing transgenes of interest were lysed in IP lysis buffer (50 mM Tris-Cl (pH 7.4) containing 150 mM NaCl, 0.5 mM DTT, 1% NP-40, 10% glycerol, 1x cOmplete protease inhibitor cocktail [Roche]). This lysate was incubated under rotation at 4°C for 1 hour and centrifuged at 4°C for 30 min at 15000 × g. Subsequently, the supernatant was taken and snap frozen in liquid nitrogen for later analyses. Lysates from cells expressing GFP- and RFP-tagged transgenes were subjected to affinity enrichment for ~3 hours at 4ºC using GFP-TRAP and RFP-TRAP beads (Chromotek), respectively. Before V5-immunopurification, lysates were pre-cleared using empty protein A/G PLUS Agarose beads (Santa Cruz) under rotation at 4°C for ~4 hours. Subsequently, V5-tagged transgenes were precipitated overnight using V5-agarose beads under rotation at 4°C. For immunoprecipitation of Ago3- and Piwi5-complexes, antibodies described in the previous section were added to lysates at 1:10 dilution and incubated ~4 hours at 4ºC. Subsequently, A/G PLUS Agarose beads (Santa Cruz) were added and bound overnight. For all IP-experiments, beads were equilibrated using lysis buffer prior to immunoprecipitation. Subsequently, beads were washed thrice in wash buffer (10mM Tris-Cl (pH 7.5), 150mM NaCl, 0.5mM EDTA, 0.1% NP-40, 1x complete protease inhibitors) and heated at 90°C for 5 minutes in 2xSDS sample buffer (120mM Tris-Cl (pH 6.8), 4% SDS, 10% β-mercaptoethanol, 20% glycerol, 0.04% bromophenol blue) to dissociate protein from the beads. Prior to western blot analyses, samples were diluted to 1x SDS sample buffer by adding an equal volume of wash buffer, resolved on 7.5% polyacrylamide gels, and blotted to nitrocellulose membranes. The following antibodies, generated in our laboratory, were used for western blotting: rabbit-anti-Ago3 (1:500) and rabbit-anti-Piwi5 (1:500). Rabbit-anti-GFP was used 1:10,000 (kind gift from F. van Kuppeveld (Utrecht University) (20). The following commercially available antibodies were used: rat-anti-GFP (1:1000, ChromoTek, 3h9, RRID:AB_10773374), rat-anti-α-tubulin (1:1000, Bio-Rad, MCA77G, RRID: AB_325003), mouse anti-flag (1:1000, Sigma, F1804, RRID: AB_262044), mouse anti-RFP (1:1000, ChromoTek, 6g6, RRID: AB_2631395), rabbit anti-sDMA (1:1000, Millipore, 07-413, RRID: AB_310595). Secondary antibodies were goat-anti-rabbit-IRdye800 [Li-cor; 926-32211, RRID: AB_621843], goat-anti-mouse-IRdye680 [926-68070, RRID: AB_10956588] and goat-anti-rat-IRdye680 [926-68076, RRID: AB_10956590] (all 1:10,000).

**Small RNA Immunoprecipitation and deep sequencing**

Aag2 cells were infected with SINV-GFP at an MOI of 1 and lysed in small RIP-lysis buffer (10mM Tris-HCl (pH 7.4), 150mM NaCl, 0.5mM EDTA, 0.1% SDS, 1% Triton X-100, 1% Sodium deoxycholate, 10% Glycerol, 1x complete protease inhibitors) 48 hours post infection. PIWI proteins were purified from cell lysates as described above. After immunoprecipitation, PIWI-piRNA complexes were washed three times in small RIP-lysis buffer. To isolate RNA, complexes were treated with 100μg proteinase K (Ambion, #AM2546) for 2 hours at 55ºC with gently mixing in proteinase K buffer (50mM Tris-HCl (pH 7.8), 50mM NaCl, 0.1% NP-40, 10mM Imidazole, 1% SDS, 5mM EDTA, 5mM β-mercaptoethanol). RNA was purified using Phenol-Chloroform (pH 4.5) seperation followed by ethanol precipitation. Purified small RNAs were cloned and sequenced using Illumina's Truseq technology.

**Mass spectrometry**

For mass spectrometry analysis, PEI-transfected Aag2 cells were lysed in IP lysis buffer, while untransfected Aag2 cells served as control. Approximately 3 mg protein lysate was subjected to GFP-affinity purification using 7.5uL GFP-TRAP beads (Chromotek) for ~1.5 hours at 4ºC. Beads were

washed stringently twice in lysis buffer, twice in PBS containing 1% NP-40, and three times in PBS, followed by on-bead trypsin digestion as described previously (21). Subsequently, tryptic peptides were acidified and desalted using Stagetips, eluted and brought onto an EASY-nLC 1000 Liquid Chromatograph (Thermo Scientific). Mass spectra were recorded on a QExactive mass spectrometer (Thermo Scientific). MS and MS2 data were recorded using TOP10 data-dependent acquisition. Raw data files were analyzed using Maxquant version 1.5.1.0. using standard recommended settings (22). LFQ, IBAQ and match between runs were enabled. Data were mapped to the *Aedes aegypti* proteome included in the NCBI *Ae. aegypti* Annotation Release 101 (released 12 July 2017). Data were further analyzed and visualized using Perseus version 1.3.0.4 (23) and R. In short, identified proteins were filtered for contaminants and reverse hits. LFQ-values were subsequently $\log_2$-transformed and missing values were imputed assuming a normal distribution. A t-test was then performed to calculate significantly enriched proteins. Volcano plots were generated with R. The mass spectrometry proteomics data have been deposited to the ProteomeXchange Consortium via the PRIDE (24) partner repository with the dataset identifier PXD009997.

**Density gradient centrifugation**

Aag2 cells stably expressing GFP-Ven were lysed in buffer containing 10 mM Hepes-KOH (pH 7.5), 50 mM Tris-HCl (pH 7.5), 150 mM KCl, 5 mM $MgCl_2$, 10% glycerol, 0.5% NP-40 and 1x cOmplete protease inhibitor cocktail [Roche]). The lysate was incubated under rotation at 4ºC for one hour followed by centrifugation at 15000 × g, 4ºC for 30 minutes to spin down debris. The supernatant was brought onto a 10-45% Sucrose gradient in 10 mM Hepes-KOH (pH 7.5), 50 mM Tris-HCl (pH 7.5), 150 mM KCl, 5 mM $MgCl_2$ and spun at 92,100 × g in a TH641 Sorvall rotor at 4ºC for 20 hours after which 24 equal fractions of 500 µL were collected. From each fraction, 100 µL was added to 700 µL acetone and 200 µL trichloroacetic acid (T0699, Sigma) and incubated on ice for 40 minutes to precipitate proteins. After centrifugation at 15000 × g, 4ºC for 30 minutes, pellets were washed twice in 1 mL acetone and resuspended in 8 M urea, 20 mM DTT. RNA was isolated from the remaining 400 µL of each fraction using acid phenol/chloroform extraction.

## SUPPLEMENTAL REFERENCES


1. Akbari, O.S., Antoshechkin, I., Amrhein, H., Williams, B., Diloreto, R., Sandler, J. and Hay, B.A. (2013) The developmental transcriptome of the mosquito Aedes aegypti, an invasive species and major arbovirus vector. *G3 (Bethesda)*, **3**, 1493-1509.
2. Liu, H., Wang, J.Y., Huang, Y., Li, Z., Gong, W., Lehmann, R. and Xu, R.M. (2010) Structural basis for methylarginine-dependent recognition of Aubergine by Tudor. *Genes & development*, **24**, 1876-1881.
3. Friberg, A., Corsini, L., Mourao, A. and Sattler, M. (2009) Structure and ligand binding of the extended Tudor domain of D. melanogaster Tudor-SN. *J Mol Biol*, **387**, 921-934.
4. Soding, J., Biegert, A. and Lupas, A.N. (2005) The HHpred interactive server for protein homology detection and structure prediction. *Nucleic acids research*, **33**, W244-248.
5. Finn, R.D., Clements, J., Arndt, W., Miller, B.L., Wheeler, T.J., Schreiber, F., Bateman, A. and Eddy, S.R. (2015) HMMER web server: 2015 update. *Nucleic acids research*, **43**, W30-38.
6. Di Tommaso, P., Moretti, S., Xenarios, I., Orobitg, M., Montanyola, A., Chang, J.M., Taly, J.F. and Notredame, C. (2011) T-Coffee: a web server for the multiple sequence alignment of protein and RNA sequences using structural information and homology extension. *Nucleic acids research*, **39**, W13-17.
7. Letunic, I., Doerks, T. and Bork, P. (2015) SMART: recent updates, new developments and status in 2015. *Nucleic acids research*, **43**, D257-D260.
8. Finn, R.D., Bateman, A., Clements, J., Coggill, P., Eberhardt, R.Y., Eddy, S.R., Heger, A., Hetherington, K., Holm, L., Mistry, J. *et al.* (2014) Pfam: the protein families database. *Nucleic acids research*, **42**, D222-D230.
9. Lancaster, A.K., Nutter-Upham, A., Lindquist, S. and King, O.D. (2014) PLAAC: a web and command-line application to identify proteins with prion-like amino acid composition. *Bioinformatics*, **30**, 2501-2502.

10. Vodovar, N., Bronkhorst, A.W., van Cleef, K.W., Miesen, P., Blanc, H., van Rij, R.P. and Saleh, M.C. (2012) Arbovirus-derived piRNAs exhibit a ping-pong signature in mosquito cells. *PLoS One*, **7**, e30861.
11. Anderson, M.A., Gross, T.L., Myles, K.M. and Adelman, Z.N. (2010) Validation of novel promoter sequences derived from two endogenous ubiquitin genes in transgenic Aedes aegypti. *Insect molecular biology*, **19**, 441-449.
12. Miesen, P., Girardi, E. and van Rij, R.P. (2015) Distinct sets of PIWI proteins produce arbovirus and transposon-derived piRNAs in Aedes aegypti mosquito cells. *Nucleic acids research*, **43**, 6545-6556.
13. Pall, G.S. and Hamilton, A.J. (2008) Improved northern blot method for enhanced detection of small RNA. *Nat Protoc*, **3**, 1077-1084.
14. Livak, K.J. and Schmittgen, T.D. (2001) Analysis of relative gene expression data using real-time quantitative PCR and the 2(-Delta Delta C(T)) Method. *Methods*, **25**, 402-408.
15. Blankenberg, D., Gordon, A., Von Kuster, G., Coraor, N., Taylor, J., Nekrutenko, A. and Galaxy, T. (2010) Manipulation of FASTQ data with Galaxy. *Bioinformatics*, **26**, 1783-1785.
16. Langmead, B., Trapnell, C., Pop, M. and Salzberg, S.L. (2009) Ultrafast and memory-efficient alignment of short DNA sequences to the human genome. *Genome biology*, **10**, R25.
17. Crooks, G.E., Hon, G., Chandonia, J.M. and Brenner, S.E. (2004) WebLogo: a sequence logo generator. *Genome research*, **14**, 1188-1190.
18. Schneider, T.D. and Stephens, R.M. (1990) Sequence logos: a new way to display consensus sequences. *Nucleic acids research*, **18**, 6097-6100.
19. Schindelin, J., Arganda-Carreras, I., Frise, E., Kaynig, V., Longair, M., Pietzsch, T., Preibisch, S., Rueden, C., Saalfeld, S., Schmid, B. *et al.* (2012) Fiji: an open-source platform for biological-image analysis. *Nature methods*, **9**, 676-682.
20. Wessels, E., Duijsings, D., Niu, T.K., Neumann, S., Oorschot, V.M., de Lange, F., Lanke, K.H.W., Klumperman, J., Henke, A., Jackson, C.L. *et al.* (2006) A viral protein that blocks Arf1-mediated COP-I assembly by inhibiting the guanine nucleotide exchange factor GBF1. *Dev Cell*, **11**, 191-201.
21. Smits, A.H., Jansen, P.W., Poser, I., Hyman, A.A. and Vermeulen, M. (2013) Stoichiometry of chromatin-associated protein complexes revealed by label-free quantitative mass spectrometry-based proteomics. *Nucleic acids research*, **41**, e28.
22. Cox, J. and Mann, M. (2008) MaxQuant enables high peptide identification rates, individualized p.p.b.-range mass accuracies and proteome-wide protein quantification. *Nat Biotechnol*, **26**, 1367-1372.
23. Tyanova, S., Temu, T., Sinitcyn, P., Carlson, A., Hein, M.Y., Geiger, T., Mann, M. and Cox, J. (2016) The Perseus computational platform for comprehensive analysis of (prote)omics data. *Nature methods*, **13**, 731-740.
24. Vizcaino, J.A., Csordas, A., del-Toro, N., Dianes, J.A., Griss, J., Lavidas, I., Mayer, G., Perez-Riverol, Y., Reisinger, F., Ternent, T. *et al.* (2016) 2016 update of the PRIDE database and its related tools. *Nucleic acids research*, **44**, D447-456.
